# Deployment of a Resuscitative Endovascular Balloon Occlusion of the Aorta Device in a Case of Gunshot Wound Injury to a Horseshoe Kidney

**DOI:** 10.7759/cureus.3399

**Published:** 2018-10-02

**Authors:** Karen Castaneda, Yana Puckett, Andres Leal, Catherine A Ronaghan

**Affiliations:** 1 Miscellaneous, Texas Tech University Health Sciences Center, Lubbock, USA; 2 Surgery, Texas Tech University Health Sciences Center, Lubbock, USA

**Keywords:** horseshoe kidney, gunshot wound, blunt abdominal trauma, reboa

## Abstract

A horseshoe kidney (HSK) is a urological malformation that is typically found incidentally after a traumatic injury due to its asymptomatic nature. We present a 25-year-old male with multiorgan injuries secondary to blunt abdominal trauma caused by a gunshot wound. We report the courses of action taken that led to the identification of the HSK and other associated intra-abdominal injuries and the subsequent surgical management. Resuscitative endovascular balloon occlusion of the aorta (REBOA) is an improving minimally invasive technique that was used to control hemorrhage in the early preoperative stages and during surgical repair of the injuries. Multiorgan injuries that involve an HSK are uncommon. Our interest in the case relies on the rarity and unique aspects of the injuries and the recovery of the patient following the use of REBOA.

## Introduction

Horseshoe kidneys (HSK) are among the most common congenital malformations of the urinary tract involving anomalies such as ectopia, malrotation, and vascular changes. They have an approximate frequency of 1:500 and are twice as likely to occur in males [1-2]. Kidneys generally appear between the fourth and the sixth week of development [3-4]. Fusion anomalies also occur during this time and can occur up to week nine of development [4-5]. Kidneys are initially located in the pelvis and ascend to their final position in the retroperitoneal renal fossa by the ninth week [4]. As they ascend, the blood supply originates from the aorta at continuously higher levels, while inferior branches degenerate [4,6]. The kidneys also rotate medially [7]. Occasionally, the kidneys can be pushed together during their ascent leading to the fusion of lower poles and the formation of an HSK [1-2]. An HSK is usually found at the level of the lumbar vertebra as its ascent is impeded by the inferior mesenteric artery [6]. However, it can be found anywhere along the normal path of ascent with the position being more ectopic as the fusion is more complete [4].

The vascular supply is variable in terms of the origin, number, and size of the renal arteries and veins. Arterial origins can come from the aorta, common iliac artery, medial sacral artery, lumbar artery, internal iliac artery, external iliac artery, and phrenic artery [2,4]. The vasculature of the isthmus is also variable. It can obtain its blood supply from the main renal artery, aorta, common iliac artery, or inferior mesenteric artery [4].

There are various factors that explain the embryogenesis of an HSK, from positional factors, anomalous fusion related to proximity, abnormalities in the migration of metanephric cells, intrauterine factors, and genetic factors [4]. The isthmus of an HSK can contain fibrous or parenchymatous tissue. The majority of HSKs, 80%, are found to have a parenchymatous isthmus that is thought to result from the abnormal migration of posterior nephrogenic cells across the primitive streak [4,7]. In this theory, the isthmus occurs due to ectopic mesenchymal tissue as opposed to the fusion of nephrogenic blastemas when they come in close contact, which results in a fibrous isthmus [7].

Findings of an HSK are typically incidental, since most, approximately 60%, are asymptomatic [3]. Their anatomical position, outside from the normal protection provided by the lower part of the ribcage, make them more susceptible to abdominal trauma. Renal injury occurs in 10% of abdominal traumas and 7% occurs in kidneys with a congenital or acquired disorder [3]. We present a renal trauma case from a gunshot wound (GSW) in a patient with an unknown HSK.

## Case presentation

**Figure 1:**
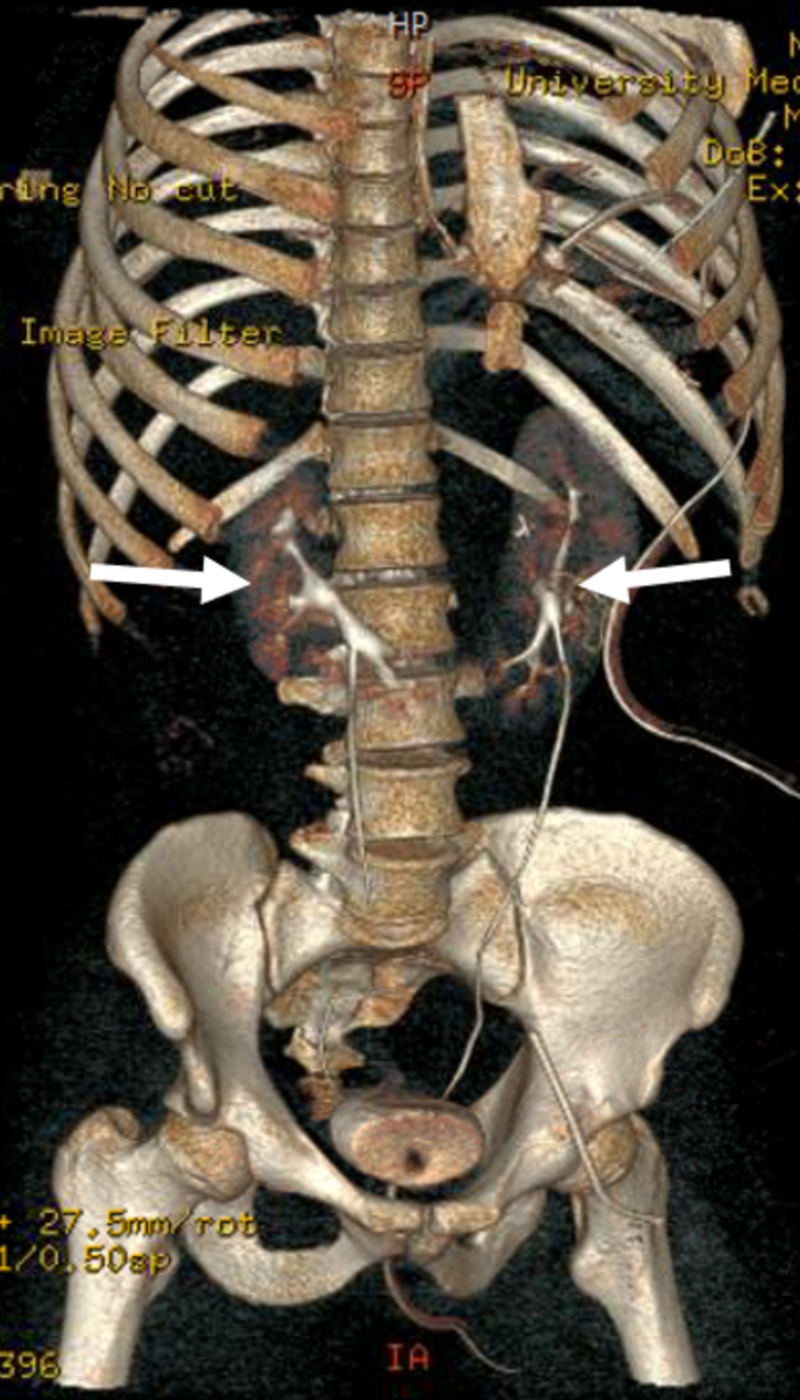
Postoperative three-dimensional view of the horseshoe kidney of the patient with gunshot wound injury and subsequent repair to the left portion of the horseshoe kidney.

A 25-year-old male presented to the emergency department (ED) at our Level I Trauma Unit after receiving a GSW in the back. The patient was found to be peritonitic and tachycardic upon physical examination in the ED triage. A focused assessment with sonography for trauma exam showed fluid in the peritoneum. A resuscitative endovascular balloon occlusion of the aorta (REBOA) was deployed in the ED for active proximal control of the hemorrhage. He was transferred to the operating room (OR) for an exploratory laparotomy, where numerous intra-abdominal injuries were found, including a splenic hilum injury, renal artery injury, and proximal bowel injury. The patient was noted to have an HSK, making the hemorrhage from the kidney more difficult to control (Figure 1). The renal capsule was opened and a 5 mm penetrating wound was found going through the left side of the HSK. Due to ongoing hemorrhage, the REBOA device was once again deployed. A large left renal vein was noted to be lacerated. This vein was ligated using 0-silk suture. The patient did well postoperatively and was discharged on postoperative Day 5.

## Discussion

Renal traumatic injuries in an unsuspected HSK are rare, especially following a GSW. Most, approximately 63%, are primarily due to motor vehicle collisions [8]. Their initial management is similar to that of any other patient presenting with blunt abdominal trauma, but special considerations regarding treatment must be taken into account. Albeit challenging to determine, early recognition is of critical importance for prognosis.

Standards of treatment for traumatic injuries tend to follow the most conservative options from observation to minimally invasive procedures to open surgical management [8-9]. Cases have been reported with successful outcomes using minimally invasive procedures such as the use of stents, while others have presented with complications that require exploratory laparotomies and open surgery [9]. The anatomical complexity of an HSK adds to the difficulty in surgical management of multiorgan injuries due to the high variability in location, orientation, and blood supply [3,9]. On account of this complexity, reports have suggested that abnormal kidneys be treated on an individual basis to focus on the patient’s clinical course to guide management [9-10].

Non-compressible hemorrhages from blunt abdominal trauma can be difficult to control using a conservative approach. The current management is highly invasive [3]. REBOA is a technique that involves the insertion of a catheter through the common femoral artery and the expansion of a balloon at a level appropriate for hemorrhage control. It is less invasive than open thoracotomies and exploratory laparotomies. There have been studies identifying REBOA as a favorite alternative such as one prospective study that compared the use of open thoracotomy and REBOA, and others in Japan that compared the use of aortic cross-clamping and REBOA [11-12]. However, the data is inconclusive, and more research is still needed to identify the effectiveness of REBOA [13]. REBOA carries a risk for irreversible organ damage, cardiac failure, and exacerbation of traumatic brain injury [14-15]. Partial REBOA (pREBOA) where the balloon is partially deflated, and intermittent REBOA (iREBOA) where the balloon is fully deflated for brief periods of time, have been developed to address these limitations. However, data on these are also limited.

## Conclusions

In our case, deployment of a REBOA device intraoperatively allowed us to visualize the injury to an HSK with ease, allowing quick repair of the injury. Overall, REBOA has been increasingly used worldwide, but further research and training are needed to ensure reliable indications and methods and establish its effectiveness.
